# Reactions of a Polyhalide Ionic Liquid with Copper, Silver, and Gold

**DOI:** 10.1002/open.201800149

**Published:** 2018-10-31

**Authors:** Benjamin May, Matthias Lexow, Nicola Taccardi, Hans‐Peter Steinrück, Florian Maier

**Affiliations:** ^1^ Lehrstuhl für Physikalische Chemie II Friedrich-Alexander-Universität Erlangen-Nürnberg Egerlandstr. 3 91058 Erlangen Germany; ^2^ Lehrstuhl für Chemische Reaktionstechnik Friedrich-Alexander-Universität Erlangen-Nürnberg Egerlandstr. 3 91058 Erlangen Germany

**Keywords:** corrosion, ionic liquids, metal dissolution, photoelectron spectroscopy, surface analysis

## Abstract

The reactions of copper, silver, and gold with the imidazolium‐based polyhalide ionic liquid (IL) [C_6_C_1_Im][Br_2_I] were investigated by using X‐ray photoelectron spectroscopy (XPS), weight‐loss measurements, and gas‐phase mass spectrometry. All three Group 11 metals are strongly corroded by the IL at moderate temperatures to give a very high content of dissolved Cu^I^, Ag^I^, and Au^I^ species. The IL–metal solutions are stable against contact with water and air. The replacement of imidazolium with inorganic sodium cations decreased metal corrosion rates by orders of magnitude. Our results clearly indicate metal oxidation by iodide from dibromoiodide anions to form molecular iodine and anionic [Br‐M^I^‐Br]^−^ (M=Cu, Ag, Au) complexes stabilized by imidazolium counterions. From experiments with a trihalide IL with imidazolium methylated at the 2‐position, we ruled out the formation of imidazole–carbene as a cause of the observed corrosion. In contrast to Group 11 metals, molybdenum is inert against the trihalide IL, which is attributed to surface passivation.

## 1 Introduction

Ionic liquids (ILs) are a quite new class of salts that are often liquid well below 100 °C. They exhibit unique physicochemical properties, such as extremely low vapor pressures, wide electrochemical windows, and unusual solvation characteristics, to name only a few, which makes them candidates for many applications.^1^ ILs are mostly comprised of organic cations and organic or inorganic anions that are covalently bound molecular entities. More unusual are ILs that are based on polyhalide anions, such as triiodide [I_3_]^−^, due to the related I^−^+I_2_⇌[I_3_]^−^ equilibrium.^2^

Polyhalide anions have a long history in aqueous systems, but have only recently been an active area of interest in ILs. New synthesis techniques have allowed for an expansion of the number of known polyhalide anions (particularly those of the lighter halides), and several interesting potential applications for polyhalide ILs have emerged:^3^ Tribromide salts have been used in organic synthesis as convenient bromination agents.^4^ Tribromide‐ and trichloride‐containing ILs exhibit enhanced reactivity and higher yields in halogenation reactions.^5^ Moreover, they also exhibit stereo‐6 and regioselectivity,^7^ and the reusability of some of these agents has been demonstrated.^6^, ^7^, ^8^ In addition, IL‐based polyhalides have great potential as “green” halogenation reagents due to their low volatility, which makes them easier to handle than the base halogens. Another application for trihalide ionic liquids is related to the electrolyte in dye‐sensitized solar cells (DSSCs). Classic DSSCs based on IL ions make use of the [I_3_]^−^/I^−^ redox couple.^9^ Recently, [Br_3_]^−^/Br^−^‐based redox couples have also come into focus in this context.^10^ Polyhalide ionic liquids are of interest as components in both liquid and quasi‐solid electrolytes.^11^ Understanding the behavior of polyhalide species in ionic liquids is, therefore, an important step in optimizing redox couple/electrolyte chemistry. Moreover, an understanding of the corrosive nature of polyhalide‐containing electrolytes when in contact with metal electrodes—often made from precious metals and/or carbonaceous electrodes decorated with metal electrocatalysts—is crucial for the long‐term stability issues of DSSC devices: it is known that iodine is corrosive to metallic grid contacts, such as silver, when water and air are present.^12^ Furthermore, wet‐chemical metal dissolution plays an important role when etching gold microstructures in microelectronic devices by using aqueous iodide/iodine trihalide systems, or when employing cyanide‐based chemistry for gold leaching, to name only two examples. The latter has the disadvantage of high toxicity and disposal issues, the former generates volatile iodine formed during the etching process (a recent review is provided by Green^13^).

One of the key properties of ILs is their low volatility, which allows for investigations under ultrahigh vacuum (UHV) conditions, such as X‐ray photoelectron spectroscopy (XPS). Over the last decade, XPS studies on a large number of nontrihalide IL systems have been successfully carried out, proving the strength of this surface‐sensitive technique (for reviews, see Steinrück^14^ and Maier et al.^15^). XPS has also recently been applied to trihalide ionic liquid systems by Men et al.^16^ By using a conventional laboratory XPS setup and imidazolium‐based [Br_2_I]^−^ and [I_3_]^−^ ILs, the authors showed that the vapor pressure was low enough to perform XPS and that beam damage effects were negligible when X‐ray exposure was restricted. By analyzing the shifts in binding energy and shake‐up loss features, Men et al. clearly showed that [Br_2_I]^−^ and [I_3_]^−^ are weakly coordinating anions with very low basicity. The negative excess charge on the anions is delocalized over the trihalide complexes to form a π system. For [Br_2_I]^−^, the authors found the anionic central iodine atom sitting in a linear [Br‐I‐Br]^−^ complex to be less electronegative than its bromine neighbors.^16^ In their XPS studies, Men and co‐workers placed small IL droplets onto a stainless‐steel sample holder. Initial XPS studies by our group to investigate similar trihalide ILs in the form of thin films spread on polycrystalline gold foils (our standard inert support) were stymied by the fact that the brownish IL films first quickly darkened and then lost color under UHV. Moreover, these IL samples on gold showed an increased background pressure compared with conventional ILs, which gave a first indication of a corrosion reaction between the IL and gold to give volatile byproducts.

To elucidate the effect of trihalide ILs on noble metals, we carried out a systematic investigation of Group 11 metals (Au, Ag, and Cu). These three metals from the same group have been chosen because they are relevant electrode‐contact materials in DSSCs and in electronic devices in general. They were exposed to two imidazolium‐based ILs with the trihalide anion dibromoiodide [Br_2_I]^−^, namely 1‐hexyl‐3‐methylimidazolium dibromoiodide ([C_6_C_1_Im][Br_2_I]) and 1‐butyl‐2,3‐dimethylimidazolium dibromoiodide ([C_4_C_1_C_1_Im][Br_2_I]). The latter was chosen to elucidate the role of potential metal carbene formation. We demonstrate that our corrosion procedure is a simple way to synthesize IL systems with a high content of gold(I), silver(I), and copper(I) species, which are stable against exposure to ambient conditions and water. In contrast to the extensive studies of transition metals, aluminum, and steel compounds corroded by ionic liquids,^17^ to the best of our knowledge this is the first systematic investigation into the etching of Group 11 metals by a trihalide ionic liquid.

## 2 Results

### 2.1 Bulk Metal Corrosion

When the copper, silver, and gold foils were immersed in the [C_6_C_1_Im][Br_2_I] IL, it was noted that the initially dark red‐brown IL turned black within minutes and became more viscous over time. This darkening process occurred much faster with copper foil than with gold and silver foils. With molybdenum foil, no color change was noted.

**Figure 1:**
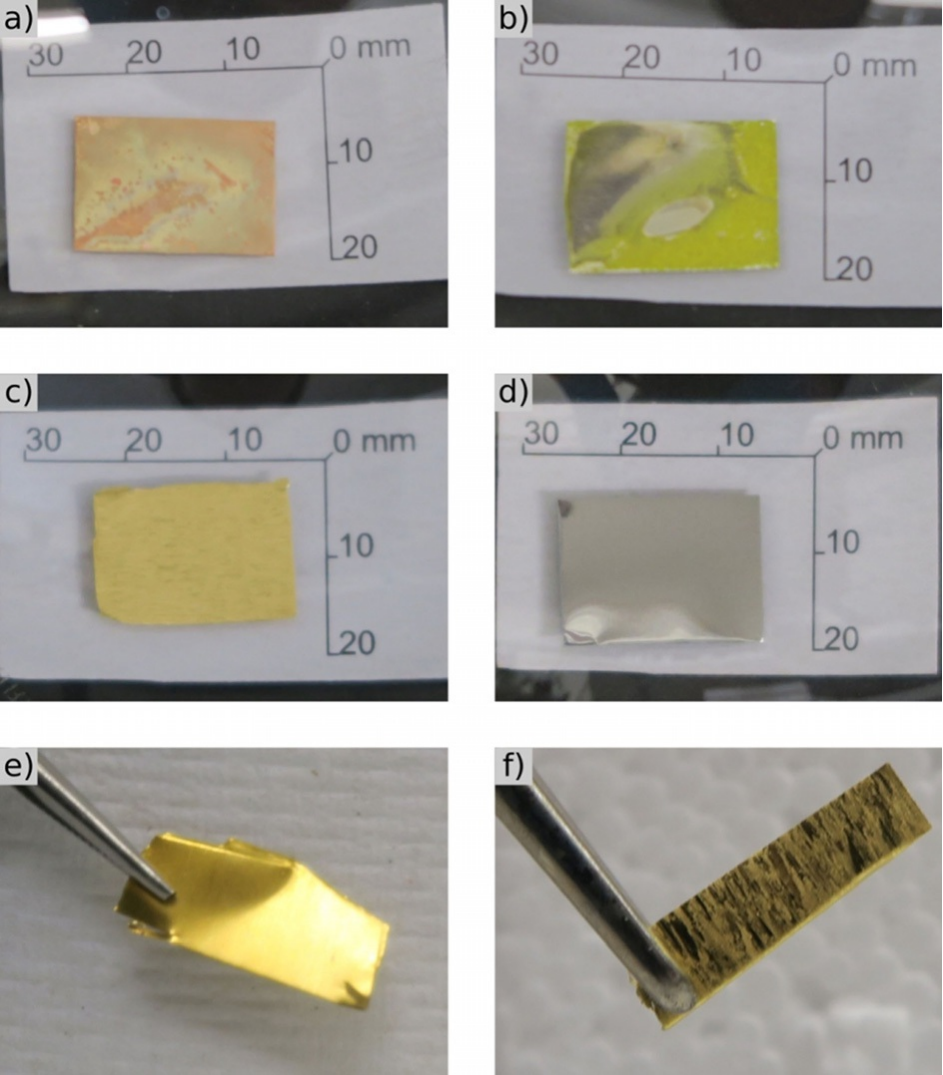
Photos of metal foils after immersion in [C_6_C_1_Im][Br_2_I] for 6 h and rinsing with acetone: a) copper, b) silver, c) gold, d) molybdenum. For direct comparison, photos of a gold foil (0.1 mm thick, ≈4×18 mm) were taken e) before and f) after corrosion under grazing illumination conditions.

All the Group 11 metal foils (gold, silver, and copper) showed considerable mass loss after exposure to [C_6_C_1_Im][Br_2_I] for 6 h at 40 °C (Table 1). Converting the metal mass loss to metal mmol, copper was dissolved to the largest extent (3.22 mmol Cu per 4.1 mmol of initial IL, that is, nominally a 0.78:1 molar ratio) compared with gold (0.30:1) and silver (0.43:1). For gold, extended exposure to the IL was tested (Au‐21h, Table 1), with the second gold foil initially exposed to [C_6_C_1_Im][Br_2_I] at 40 °C for about 11 h, then left in the IL without heating for a further 10 h. The slightly increased mass loss of gold after extended exposure showed that maximum gold dissolution had not been reached after 6 h; however, the 6 h exposure will be discussed here in most cases for consistency. In contrast to the Group 11 metals, molybdenum showed virtually no mass loss. Visual inspection of the metals before and after corrosion (Figure 1) revealed clear changes in the copper, silver, and gold foils: instead of a shiny smooth surface, tarnished patches were seen on copper and silver. The apparent greenish patina on the copper is consistent with the formation of copper halide salts. Gold did not show any color changes, but was clearly etched; the elongated grain structure typical of a rolled polycrystalline foil became apparent after immersion in the trihalide IL. To obtain a better microscopic view, a 0.1 mm thick gold foil and a 1 mm thick gold wire were partly immersed in [C_6_C_1_Im][Br_2_I] for about 17 h at room temperature, then scanning electron microscopy (SEM) pictures were recorded after the samples were rinsed with acetone (see Figure S1 in the Supporting Information). Whereas the unexposed part of the foil revealed a smooth and flat appearance, the immersed part displayed a rough surface with etched grooves and pits. Moreover, the exposed Au wire clearly revealed a 6 % decrease in diameter, and the formerly smooth surface became rough and decorated with etching pits on the μm scale (Figure S1). In contrast to the Group 11 metals, molybdenum exhibited no visual changes in the surface; the negligible mass loss and the unchanged surface appearance both indicate that no corrosion of the molybdenum foil took place.

**Table 1:** Change in mass (and in amount of substance) of the metal foils after immersion in [C_6_C_1_Im][Br_2_I] at 40 °C for 6 h. In case of Au‐21 h, the Au foil was kept in the IL at 40 °C for 11 h, then left for a further 10 h at room temperature. Mass losses are also given in dissolution / etching rates commonly used in literature. Moreover, metal contents in the remaining IL solutions as derived from mass loss after corrosion are given in molar ratio (mol metal loss : mol initial IL) and in molar content.

	Initial [mg]	Final [mg]	Δ [mg]	Δ [mmol]	Dissolution rate [g dm^−2^ d^−1^]	Etch rate [μm min^−1^]	IL [mg]	IL [mmol]	Metal/IL molar ratio	Metal [mol %]
Au‐6h	480	245	235	1.19	18.9	0.068	1783	3.93	0.30:1	23
Au‐21h	528	198	330	1.68	6.89	0.025	1935	4.26	0.39:1	28
Ag‐6h	384	196	188	1.74	12.3	0.082	1854	4.08	0.43:1	30
Cu‐6h	267	63	204	3.22	13.7	0.106	1874	4.13	0.78:1	44
Mo‐6h	329	328	1	≈0	≈0	≈0	1998	4.40	0.00:1	0

From the mass losses summarized in Table 1, it is clear that the three Group 11 metals are susceptible to considerable bulk corrosion by [C_6_C_1_Im][Br_2_I], which leads to an IL solution with a metal content well above 20 mol %. For comparison with common dissolution rates, such as metal mass loss per exposed surface area per unit time [mg dm^−2^ d^−1^] and changes in foil height per unit time [μ min^−1^], the corresponding values are also given in Table 1. Note in the case of the most noble metal, gold, the etching rate for 6 h is of the same order of magnitude as typical wet‐chemical etching rates of 0.1 to 1 μm min^−1^ in aqueous I_2_/I^−^ or alkaline cyanide etching processes.^13^

Due to the high metal contents in the IL solutions obtained after dissolution, metal core‐level signals were expected to be clearly detectable XPS. After the 6 h corrosion experiments, the solutions were placed onto a molybdenum XPS sample holder and transferred to the XPS system. The chemical nature of the dissolved metal species, as deduced by using XPS, will be discussed in detail in Section 2.2. The concentration values derived from XPS intensities were directly compared with the mass‐loss values shown in Table 1; the quantification of metal content is discussed below. For quantification, the measured XPS intensities were converted into number of atoms (Table 2), taking the corresponding atomic sensitivity factors (ASF) into account. The signals of all atoms were normalized to the nitrogen signal that originated solely from the imidazolium cation (that is, two nitrogen atoms per cation). For the Group 11 metals, metal‐to‐imidazolium ratios of between 0.4 and 0.5 to one were found in the XPS spectra, whereas no molybdenum signals were found for the Mo sample, which confirmed the negligible corrosion of this metal. In Table 2, the reduced content of trihalide iodide is notable, particularly in the copper corrosion experiment. Apparently, the corrosion of Group 11 metals goes along with a loss of iodide. Note that apart from the IL and metal signals, minor amounts of oxygen and an excess of carbon were also detected; these findings are discussed in Section 2.2.

**Table 2:** The composition of [C_6_C_1_Im][Br_2_I] before and after the bulk corrosion experiments (6 h at 40 °C; see Table 1). The nominal composition of [C_6_C_1_Im][Br_2_I] is also given. Metal/Im denotes the ratio of metal cations to imidazolium cations.

	C 1s	O 1s	N 1s	I 3p_5/2_	Br 3d	Cu 2p_1/2,3/2_	Ag 3d_3/2,5/2_	Au 4f_5/2,7/2_	metal/Im
ASF	0.208	0.599	0.364	5.740	0.551	8.69	4.460	3.756	
nominal	10	0	2	1	2	0	0	0	
neat [Br_2_I]^−^	11.7	0.2	≡2	0.5	1.7	–	–	–	
Cu‐6h	14.4	1.2	≡2	<0.1	2.2	0.5	–	–	0.5
Ag‐6h	16.5	1.3	≡2	0.3	1.9	–	0.4	–	0.4
Au‐6h	15.2	0.6	≡2	0.5	2.1	–	–	0.4	0.4
Mo‐6h	17.3	1.4	≡2	0.7	2.0	–	–	–	0.0

The mass losses of the foils after immersion for 6 h in the polyhalide IL, and the quantification of the XPS signals of the remaining solutions, consistently revealed that, for gold, silver, and copper, a considerable amount of metal was dissolved into the IL, whereas molybdenum was virtually unaffected. The XPS spectra of the neat trihalide IL [C_6_C_1_Im][Br_2_I] and the solutions after the corrosion experiments will be discussed below in detail.

### 2.2 XPS Spectra of Neat [C_6_C_1_Im][Br_2_I] Before and After Cu, Ag, Au, and Mo Dissolution

**Figure 2:**
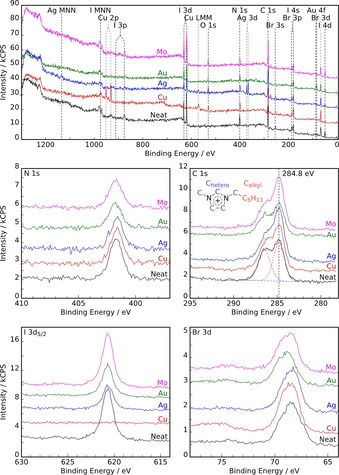
Wide (top) and detailed XP spectra of the neat [C_6_C_1_Im][Br_2_I] IL (black) and of the solutions obtained after exposure of molybdenum (magenta), gold (green), silver (blue), and copper (red) foils to [C_6_C_1_Im][Br_2_I] for 6 h at 40 °C. All spectra are referenced to aliphatic carbon at 284.8 eV.

**Figure 3:**
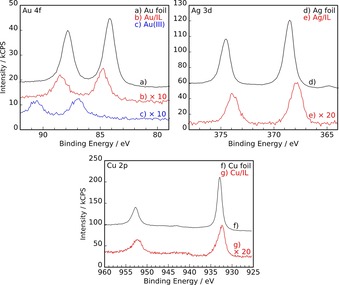
Metal core level regions of the IL solutions (red spectra) after corrosion of b) gold (Au/IL), e) silver (Ag/IL), and g) copper (Cu/IL) foils for 6 h at 40 °C in [C_6_C_1_Im][Br_2_I]. For comparison, the metal signals of freshly sputtered a) Au, d) Ag, and f) Cu foils before corrosion are shown in black along with the Au^III^ signal (c, blue trace) of the tetrabromoaurate [C_2_C_1_Im][Au^III^Br_4_] compound. For details, see the main text.

As described above, [C_6_C_1_Im][Br_2_I] quickly changed color from dark brown to black on contact with the Group 11 foils in the corrosion experiments. The black solutions were spread onto a flat XPS molybdenum sample holder and placed in the fast entry load lock of a UHV system overnight (for details, see the Experimental Section). During degassing under vacuum, the films lost considerable color to give a more‐or‐less transparent liquid layer on the surface of the sample holder the following day. This change in color from dark black to a fully clear film was most pronounced for the solution from the copper corrosion experiment, in which the most metal was dissolved due to the high etching rate (see Section 2.1.). Survey XPS spectra and IL‐ and metal‐related detailed core‐level scans are shown in Figures 2 and 3 for the neat degassed [C_6_C_1_Im][Br_2_I] trihalide IL and for the IL solutions after corrosion of the gold, silver, copper, and molybdenum foils for 6 h. The C_alkyl_ peak at 284.8 eV served as an internal reference for the binding energies, as previously described.^18^

Figure 2 shows the survey spectra and the detailed IL‐related core‐level spectra. In the C 1s region, two deconvoluted peaks originate from the five terminal carbon atoms of the alkyl chain at 284.8 eV (C_alkyl_) and from the five carbon atoms directly attached to a nitrogen atom at 286.5 eV (C_hetero_). The integrated areas of the two carbon signals of the neat IL revealed a C_alkyl_/C_hetero_ ratio of 1.1:1.0, which matches the expected 5:5 ratio to within the accuracy of our method (±10 %). The anion‐related signals of the neat IL showed a single iodide peak in the I 3d_5/2_ region at 620.7 eV, and in the Br 3d_3/2,5/2_ region at around 68.5 eV (note that, due to the small spin‐orbit‐splitting of the Br 3d signals, the binding energy values refer to the peak maxima). Notably, the I 3d_5/2_ and the Br 3d signals of [C_6_C_1_Im][Br_2_I] are considerably shifted to higher binding energies (by +2.4 and +1.1 eV), compared with the analogous monohalide ILs [C_6_C_1_Im]I and [C_6_C_1_Im]Br, respectively (Figure S2). These shifts to higher binding energy are mainly related to the delocalization of negative charge over the trihalide anion compared with the single halide anions, and have already been discussed by Licence and co‐workers.^16^ Based on the XPS binding energy shifts and Kamlet–Taft solvent parameter measurements, the authors showed that the trihalide [Br_2_I]^−^ anion is an extremely weakly coordinating anion with very low basicity. In our measurements, this is reflected by the larger binding energy separation of the C_alkyl_ and C_hetero_ peaks for [C_6_C_1_Im][Br_2_I] compared with the [C_6_C_1_Im]Br and [C_6_C_1_Im]I monohalide ILs (see the C 1s spectra in Figure 2 and Figure S2). A detailed explanation of the significance of this separation is given in Ref. 19. In short, a more strongly coordinating anion, such as bromide, allows for greater partial electron transfer to the imidazolium ring and leads to a lower positive charge and, therefore, a shift in the C_hetero_ peak towards lower binding energies. Thus, the large C_hetero_–C_alkyl_ peak separation found for [C_6_C_1_Im][Br_2_I] confirms the low coordination strength of the [Br_2_I]^−^ anion, as already proposed by Men et al.^16^

Quantification of the XPS intensities of neat [C_6_C_1_Im][Br_2_I] led to an overall good agreement with the nominal composition (see also Table 2), apart from the iodine content, which was found to be too low. For the neat IL, an atomic composition of iodine/bromine/nitrogen of 0.5:1.7:2.0 was found instead of the expected 1:2:2 (for Mo‐foil corrosion, a slightly higher iodine content in an atomic ratio of 0.7:2.0:2.0 was found). We attribute this, at least partly, to some uncertainty in the atomic sensitivity factor used here for the I 3d_5/2_ core‐level quantification (note that a I/N ratio of 0.85:2.0 was found by XPS quantification for [C_6_C_1_Im]I). Note that we derived the iodine intensity only from the main I 3d_5/2_ line, which includes the broad shake‐up shoulder around 626 eV that contains some intensity from the trihalide anions due to their delocalized charge distribution.^16^ Because the focus of this work is metal corrosion, more detailed investigations must be carried out in the future to elucidate this aspect.

The XPS spectra after the 6 h corrosion experiment with Mo foil showed no Mo signals (e.g. Mo 3d at around 230 eV), which corroborates the negligible mass loss found for the IL‐exposed Mo foil (see Table 1). The only changes were a moderate decrease in the [C_6_C_1_Im][Br_2_I] signals, along with an increase in the C_alkyl_ signal and the appearance of an O 1s peak at 530 eV. These additional C_alkyl_ and O signals are attributed to the appearance of nonvolatile contaminants in the near‐surface region after the corrosion experiment, which possibly originate from residual glassware contamination and/or adsorption from lab air. Such contaminants have been reported to be surface‐active^20^ and, therefore, show up in XPS spectra even at extremely low bulk concentrations.

Next, we discuss the IL‐derived XPS spectra in Figure 2 after the 6 h corrosion experiments of gold, silver, and copper foils at 40 °C. The most striking difference with molybdenum is the reduction in I 3d intensity (see the I 3d_5/2_ region in Figure 2 and Table 2). For the Au and Ag solutions, the I 3d_5/2_ intensity decreased by 40 and 50 %, respectively, compared with the signal in neat [C_6_C_1_Im][Br_2_I]. For copper, for which the amount of metal dissolution was found to be the highest (Table 1), the I 3d intensity was almost entirely absent. It is thus evident that during the reaction of the Group 11 metals with the IL, iodide from [Br_2_I]^−^ is consumed. Because no precipitate was found, a neutral iodine‐containing species is apparently formed that desorbs under vacuum conditions. The Br 3d intensity did not decrease (and, therefore, no neutral IBr had formed), so it is most likely that molecular iodine I_2_ was pumped away. After testing in a separate UHV system, gas‐phase mass spectra indeed showed the release of I_2_ from [C_6_C_1_Im][Br_2_I] when the IL was in contact with copper (see Figure S3). It is important to note that the Br 3d peaks given in Figure 2 did not exhibit significant changes in binding energy or peak shape before and after exposure to Cu, Ag, or Mo; only after Au corrosion were the Br 3d signals slightly shifted by +0.3 eV to higher binding energy. Thus, the formation of free bromide (Br^−^) during corrosion was ruled out due to the absence of single‐halide Br^−^ signals in the Br 3d region at lower binding energies.

Direct evidence for the dissolution of Au, Ag, and Cu was obtained from the survey spectra in Figure 2 and the detailed metal spectra in Figure 3. Compared with molybdenum, for which no metal signals could be detected in the IL, the XPS spectra of the IL solutions after corrosion experiments with Au, Ag, and Cu clearly showed the corresponding metal signals. The detailed spectra in Figure 3 cover the most prominent metal core levels, that is, Au 4f_5/2,7/2_ between 92 and 79 eV, Ag 3d_3/2,5/2_ between 388 and 364 eV, and Cu 2p_1/2,3/2_ between 960 and 925 eV. For comparison, the XPS spectra of Au^0^, Ag^0^, and Cu^0^ in the form of freshly sputtered metal foils are also depicted (Figure 3).

Both the Au^0^ reference and the dissolved Au species show the typical spin‐orbit split Au 4f_5/2,7/2_ doublet. Whereas the Au 4f_7/2_ level of the Au foil, that is, Au^0^, is found at 84.1 eV, in the IL solution after corrosion it is shifted to higher binding energy by 0.7 eV to 84.8 eV. This binding energy position is consistent with an Au^I^ species.^21^ To rule out the presence of Au^III^ in the solution, we also measured the tetrabromoaurate IL analogue [C_2_C_1_Im][Au^III^Br_4_], which showed a much higher shift of 2.8 eV to higher binding energy, that is, to 86.9 eV (see Figure 3). Notably, the widths of the Au 4f signals of the gold foil and of the IL solution are virtually identical, which is an indication that the Au^I^ metal ion in solution is present in a uniform chemical environment.

Clean silver foil exhibits a sharp Ag 3d doublet, with the Ag 3d_5/2_ level at 368.6 eV. In the IL solution after corrosion of the silver foil, a shift in the Ag 3d_5/2_ level to lower binding energy by 0.7 eV (to 367.9 eV) with no loss features was observed, which is indicative of the formation of Ag^I^.^22^ Similarly, the Cu 2p doublet of the solution was also shifted to lower binding energies by 0.6 eV (Cu 2p_3/2_ at 932.3 eV) with respect to clean copper foil (Cu 2p_3/2_ at 932.9 eV). The magnitude of the shift to lower binding energies and the absence of pronounced shake‐up structures—which should be present for a Cu^II^ species—are fully in line with the formation of a Cu^I^ species.^23^

### 2.3 Additional Gold‐Foil Corrosion Experiments

As shown above, a considerable amount of metal in the form of Au^I^, Ag^I^, and Cu^I^ species was dissolved in the trihalide IL at the expense of iodine. Whereas Ag^I^ and Cu^I^ species are stable oxidation states in aqueous systems (i.e. they form stable precipitates with dissolved halide anions), Au^I^ is metastable and prone to disproportionation to Au^III^ and Au^0^ in water without stabilizing ligands.^24^ Moreover, gold is the most noble—and, therefore, most unreactive—metal in this series. Thus, further gold‐foil corrosion experiments were carried out to elucidate 1) the effect of additional water, 2) the role of the proton in the 2‐position of the imidazolium ring, and 3) the replacement of the imidazolium cation by an inorganic cation. The results of these experiments are summarized below.

When the gold‐corrosion experiment was performed under anhydrous and water‐saturated conditions for 6 h at 40 °C, we found no significant differences in the amount of dissolved Au, and also no significant difference in the XPS spectra (see Table S1 and Figure S4) compared with the results described above. This indicates that neither water nor oxygen are necessary for (or inhibitory of) the corrosion of gold in contact with [C_6_C_1_Im][Br_2_I].

Stable Au^I^‐imidazole‐carbene complexes are known in the literature, for example, in the context of Au^I^ homogeneous catalysis.^25^ To rule out the possibility that the corrosion process found here requires or involves the formation of a stable Au^I^‐carbene (which most likely would occur by abstracting the most acidic proton at the 2‐position of the imidazolium ring), we measured the ^1^H and ^13^C NMR spectra of the IL before and after Au corrosion. These NMR spectra did not reveal any significant changes due to the gold corrosion. As an example, we show the corresponding ^1^H and ^13^C NMR spectra in Figures S5 and S6, respectively. In particular, the signals of the most acidic 2‐proton at *δ*=7.8 ppm and of the 2‐carbon at *δ*=137 ppm did not decrease, which would be the case in carbene formation. Additionally, we tested a trihalide homologue, [C_4_C_1_C_1_Im][Br_2_I], that was methylated at the 2‐position to prevent proton abstraction. Due to the higher melting point of this IL, we carried out the corresponding corrosion experiment at 50 °C, and the foil mass loss results are given in Table S1. The mass loss of gold after 6 h was about 30 % less than with the nonmethylated IL, which might be related to the higher viscosity of [C_4_C_1_C_1_Im][Br_2_I] even at 50 °C; the molar amount of gold dissolved per mole of [C_4_C_1_C_1_Im][Br_2_I], however, was independent of the methylation. Overall, the efficiency of Au corrosion was found to be very similar for [C_6_C_1_Im][Br_2_I] and [C_4_C_1_C_1_Im][Br_2_I].

Finally, we explored whether the trihalide anion was able to dissolve gold without the presence of an imidazolium counterion. For this purpose, a saturated solution of an aqueous Na[Br_2_I] system was prepared by dissolving NaBr (25 mmol) and IBr (13 mmol) in water (3.2 mL; note that excess Br^−^ was provided to stabilize Au^I^ against disproportionation into Au^0^ and Au^III^). A gold foil (305 mg) was then immersed into this highly concentrated solution for 24 h at room temperature. After rinsing and drying the remaining gold foil, we found a mass loss of (12±1) mg, that is, about 0.06 mmol Au dissolved, which proved that gold is moderately soluble under these circumstances. Note that about 1 mL of [C_6_C_1_Im][Br_2_I] or [C_4_C_1_C_1_Im][Br_2_I] is able to dissolve at least ten times more gold within 6 h.

## 3 Discussion

We have consistently shown by using weight loss and XPS experiments that the trihalide ILs [C_6_C_1_Im][Br_2_I] and [C_4_C_1_C_1_Im][Br_2_I] are able to dissolve the Group 11 metals copper, silver, and gold efficiently by forming +1 metal ions at the expense of iodine; this is in contrast to the situation for molybdenum, which is not attacked. Based on these experimental results, the following reaction mechanism of a Group 11 M^0^ metal with an [Br_2_I]^−^ anion is highly plausible:

$$M{}^{0}+\left[\mathrm{Br}\text{-}I\text{-}\mathrm{Br}\right]{}^{-}\to \left[\mathrm{Br}\text{-}M{}^{I}\text{-}\mathrm{Br}\right]{}^{-}+1/2\;I{}_{2}$$

in which the iodide of the trihalide anion oxidizes the metal atom to oxidation state +1. The reduced iodine radical eventually recombines with another iodine radical, which leads to dissolved iodine I_2_ that is later removed under vacuum conditions due to its high vapor pressure. We propose that the M^I^ ion is stabilized by two Br^−^ anions arranged in a linear fashion to form a [Br‐M^I^‐Br]^−^ anionic complex, which is known, for example, in Au^I^–halide systems.^25^

This reaction mechanism explains the XPS metal signals of oxidation state +1 in solution, the selective decrease in the iodine signals in the XPS spectra and the increase in iodine in the gas phase over the course of the reaction. We attribute the color change of the trihalide ILs (from deep brown to black) upon immersion of the Cu, Ag, and Au foils to the formation of I_2_ that remains dissolved under ambient pressure, but is lost under vacuum. The latter process was revealed by gas‐phase analysis and by the decoloration of the IL film on the sample holder after degassing. According to the proposed reaction mechanism, the amount of Br remains essentially constant, as confirmed by using XPS. The fact that the Br 3d XPS signals from the trihalide IL before and after corrosion do not exhibit new signals at different binding energy—which should be visible through significant peak shape changes—clearly supports the proposed idea that the Br atoms remain in an anionic [Br‐M^I^‐Br]^−^ complex with the negative charge delocalized. The formation of free bromide Br^−^ and free iodide I^−^ anions can be ruled out by using XPS because of their very different binding energy positions.

For the most noble metal, Au, experiments with both a carefully dried IL and in the presence of water, showed no significant difference in the corrosion efficiency, which ruled out any significant influence of water. Moreover, the similar corrosion rate of gold by [C_4_C_1_C_1_Im][Br_2_I] compared to the nonmethylated [C_6_C_1_Im][Br_2_I] IL and the lack of significant spectral changes in the cation‐related XPS peaks revealed that the imidazolium C2 proton is not involved. This conclusion was confirmed by the NMR spectra of the [C_6_C_1_Im][Br_2_I] IL after gold corrosion, which showed no indication of an Au^I^–carbene complex. Finally, the poor ability of highly concentrated aqueous Na[Br_2_I] to corrode metallic gold indicated that the imidazolium cation is not an innocent counterion, but plays an important role in the dissolution of the anionic [Br‐M^I^‐Br]^−^ complex. In particular, the fact that the trihalide ILs oxidized gold to a stable +1 state, even in the presence of excess water, is worth noting. Au^I^ is known to be relatively unstable in aqueous solution unless there is a sufficient concentration of halide ions and a low enough pH to maintain a stable complex, and will otherwise disproportionate to Au^0^ and Au^III^.^26^ It is tempting to speculate that there is a stabilizing solvation shell provided by the imidazolium cations around the [AuBr_2_]^−^ complex, which makes the imidazolium trihalide IL suitable for the corrosion process. Here, further experimental evidence, such as crystal‐structure investigations and tests with other IL cations, is needed, but is, however, out of the scope of this study.

The lack of a mass loss of molybdenum in our corrosion experiments, the lack of Mo signals in XPS spectra, and the lack of volatile I_2_ in the gas phase clearly demonstrate that the less‐noble metal molybdenum is virtually inert against attack by the trihalide IL. Most likely, the surface of this refractory metal becomes rapidly passivated on contact with the trihalide IL, which prevents further dissolution. To elucidate the nature of molybdenum passivation, more experiments are foreseen in the future.

## 4 Conclusions

We believe that our study could be quite relevant for future applications. The most obvious ones would be to use the investigated trihalide ILs as an etching medium for microfabrication, and even as a leaching medium to dissolve precious Group 11 metals from metal waste, for example, from electronic devices. One could also envisage recovering the metals by using appropriate electrochemical processes. Due to their low vapor pressure, the handling of trihalide ILs promises to be safer than oxidizing halogens or the use of CN‐based water chemistry. Finally, the formation of stable and water‐insensitive Au^I^ complexes in an IL medium may also open new possibilities for Au^I^ catalysis.

## Experimental Section

### IL Synthesis

The RT trihalide IL [C_6_C_1_Im][Br_2_I] was prepared by mixing 1‐hexyl‐3‐methylimidazolium bromide ([C_6_C_1_Im]Br, purity 99 %, purchased from IoLiTec and used without further processing, highly viscous liquid at RT) with iodine monobromide (IBr, Sigma–Aldrich, 98 %) in a 1:1 molar ratio. After mixing both components, we obtained a deep red‐brown liquid with a considerably lower viscosity than [C_6_C_1_Im]Br. We avoided exposure of the IL to daylight to prevent any photochemical reactions that might occur.

To investigate whether carbene formation played a role in the reaction, we studied a noncarbene‐forming trihalide counterpart, [C_4_C_1_C_1_Im][Br_2_I], methylated at the 2‐position of the imidazolium ring. This IL was prepared by mixing dry 1‐butyl‐2,3‐dimethylimidazolium bromide (1.314 g, 5.634 mmol; [C_4_C_1_C_1_Im]Br, 99 %, IoLiTec, used without further processing, m.p. ≈90 °C) with IBr (1.175 g, 5.681 mmol), that is, a 1:1 molar ratio. When the two ingredients were mixed in a mortar, the IL initially liquefied and a deep red‐brown liquid was obtained. The mixture was then placed in an evacuated bell jar to remove adsorbed water from the hydrophilic compound, which led to solidification after several minutes under vacuum. After regrinding, the obtained deep‐orange powder exhibited a melting point in the range of 40–50 °C.

1‐Ethyl‐3‐methylimidazolium tetrabromoaurate ([C_2_C_1_Im][AuBr_4_]) was synthesized as an IL‐analogous Au^III^ compound, which served as an XPS binding energy reference: AuBr_3_ (454.6 mg, 0.98 mmol; Sigma–Aldrich, 99.9 %) and [C_2_C_1_Im]Br (193.7 mg, 1.01 mmol; Sigma–Aldrich, >97 %) were placed in a 50 mL 2‐neck flask fitted with a septum and a reflux condenser. Dry CH_3_CN (20 mL) was added through the septum using a syringe, and the initial suspension was heated at reflux overnight. After cooling, the deep‐red solution was syringe‐filtered (0.45 μm, cellulose) and transferred to a 50 mL flask. The solvent was evaporated by using a rotary evaporator to give a dark‐red solid (yield: 616.8 mg, 95.1 wt % of original mass). The material was analyzed using inductively coupled plasma optical emission spectrometry (ICP‐OES), which revealed an Au content of 30.3 wt % (nominal: 31.4 wt %).

### Metal Foil Corrosion

To investigate the effect of the trihalide ILs on Group 11 metals, 15×20 mm foils of polycrystalline copper (0.1 mm thickness, MaTecK, purity >99.99 %), silver (0.125 mm, Chempur, 99.9 %, pre‐treated with fine silicon carbide paper), gold (0.1 mm, MaTecK, >99.99 %), and molybdenum (0.1 mm, MaTecK, 99.95 %) were rinsed with acetone, dried, weighed, and then immersed in [C_6_C_1_Im][Br_2_I] (≈1 mL) in a glass tube under air. The tubes were closed and then heated to 40 °C for 6 h in a water bath. A second gold foil (619.8 mg) was also corroded in [C_4_C_1_C_1_Im][Br_2_I] by using the same procedure, except that the reaction was carried out at 50 °C due to the higher melting point of the C2‐methylated IL. For each corrosion experiment, the boiling tubes were gently shaken occasionally. After 6 h, a few drops of the liquid were removed from the tubes and placed on a molybdenum sample holder to form a thin layer for XPS analysis. The metal foils were removed from the tubes, rinsed with acetone, dried, and reweighed. In addition, a 0.1 mm thick gold foil and a 1 mm thick gold wire were half‐immersed into [C_6_C_1_Im][Br_2_I] at RT for about 17 h. These samples were also studied by using SEM.

Two further corrosion experiments were performed with “dry” [C_6_C_1_Im][Br_2_I], and “wet” [C_6_C_1_Im][Br_2_I] to elucidate the role of water in the gold corrosion process: In the dry IL experiment, [C_6_C_1_Im][Br_2_I] (2.072 g, 4.56 mmol) was dried by heating to 50 °C in a continuously evacuated Schlenk flask for 24 h. A gold foil (609 mg) was added and the vessel was purged with nitrogen (Linde, 5.0) before being sealed against air contact. For the wet IL experiment, the IL (1.933 g, 4.26 mmol) was added to a Schlenk flask, followed by deionized water (543 mg, 30.1 mmol, 0.5 mL). The two liquids were not miscible even after several minutes of agitation, and the water formed a layer above the IL. This water layer initially had a slight red‐brown color. A gold foil (526 mg) was then placed into the bilayered system and fully immersed in the IL phase. Both Schlenk flasks were held in a water bath at 40 °C for 6 h. Samples of the ILs were taken for XPS analysis after the reaction, and the masses of the remaining gold foils were measured.

### XPS Analysis

Before and after the reactions, XPS analysis was performed for all IL samples. They were placed on a precleaned molybdenum sample holder by spreading a thin liquid film (≈0.1 mm thick) at the bottom of a reservoir (dimensions 14×20×0.5 mm) milled into the sample holder. The sample holder was introduced into a fast‐entry load lock (base pressure 5×10^−7^ mbar) and pumped down overnight before being transferred to the main UHV system for XPS analysis (base pressure better than 1×10^−10^ mbar).

In contrast to most IL samples, which were measured at RT, the spectra with the IL [C_4_C_1_C_1_Im][Br_2_I] were collected at 45 and 55 °C. The higher temperatures were necessary to melt these IL samples to avoid charging. A type K thermocouple was used to monitor the temperature of the heated samples, with a thermocouple controller to regulate the voltage of the heating filament. During measurements, the relative temperature was maintained to within ±0.1 °C; absolute sample temperature values were within ±5 °C.^27^

In the XPS system, the radiation source was a non‐monochromated Al_Kα_ anode of a Specs XR‐50 dual‐anode source, operated at 12 kV and a 20 mA emission current. All spectra were recorded in normal emission to integrate over a maximum probing depth (7–9 nm, depending on the kinetic energy^20^, ^28^) by using a Scienta R3000 concentric hemispherical analyzer in constant pass energy mode with 200 eV pass energy for survey spectra and 100 eV for detailed spectra. For the latter, the overall instrumental energy resolution was 0.9 eV. The collected data were analyzed using CasaXPS and charge‐corrected, such that the aliphatic carbon peak was at 284.8 eV (for more details on the XPS system, see Ref. 18). Note that for the systems studied here, only minor charge corrections, of the order of ±0.1 eV, had to be applied.

Peaks in the XPS spectra associated with nonmetal elements were fitted with a pseudo‐Voigt profile with a 30 % Lorentzian contribution. Doublets due to spin‐orbit splitting (such as I 3d) were fitted with two peaks constrained to have equal full width at half maxima (FWHM) and the expected peak‐area ratio (e.g., 2:3 for 3d_3/2_/3d_5/2_). Metal peaks were fitted similarly, except the pseudo‐Voigt profiles had an 80 % Lorentzian contribution. Shirley backgrounds were used. Carbon 1s spectra were fitted with a two‐peak model, C_alkyl_ and C_hetero_. The C_hetero_ peak was constrained to a FWHM of 1.1 times the width of the C_alkyl_ peak.

## Conflict of interest

*The authors declare no conflict of interest*.

## Supporting information

As a service to our authors and readers, this journal provides supporting information supplied by the authors. Such materials are peer reviewed and may be re‐organized for online delivery, but are not copy‐edited or typeset. Technical support issues arising from supporting information (other than missing files) should be addressed to the authors.

Supplementary
