# Supplementary material for: Reactions of a Polyhalide Ionic Liquid with Copper, Silver, and Gold
Source: ChemistryOpen. 2018 Oct 31;8(1):15–22. doi: 10.1002/open.201800149 (PMC6317927; doi:10.1002/open.201800149)
Supplement: Supplementary file 1 — Supplementary [file OPEN-8-15-s001.pdf]

ChemistryOPEN

Supporting Information

© 2018 The Authors. Published by Wiley-VCH Verlag GmbH & Co. KGaA, Weinheim

Reactions of a Polyhalide Ionic Liquid with Copper, Silver, and Gold

Benjamin May,[a] Matthias Lexow,[a] Nicola Taccardi,[b] Hans-Peter Steinrück,[a] and Florian Maier*[a]

open_201800149_sm_miscellaneous_information.pdf

Scanning electron microscopy of gold before and after exposure to [C_6C_1Im][Br_2I]

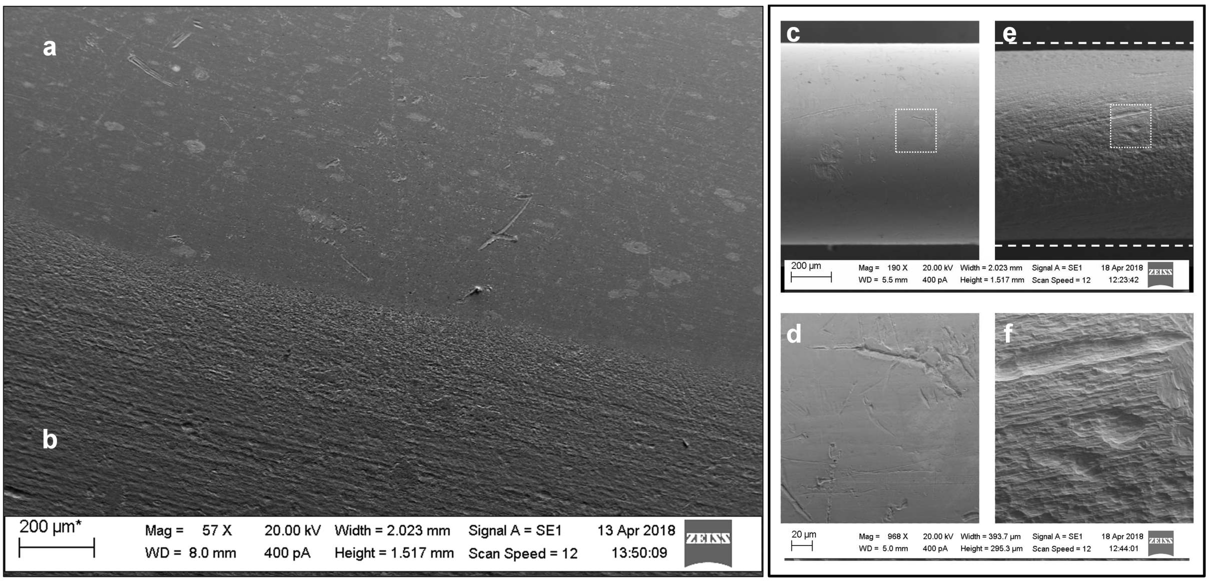

Figure S1 Scanning electron microscope (SEM) pictures of a 0.1 mm gold foil and a 1.0 mm thick gold wire, both simultaneously immersed in [C_6C_1Im][Br_2I] for 17 hours at room temperature in air.

Left: Upper half (a) unexposed area of gold foil in direct comparison with immersed bottom half (b). Right: Gold wire before (c, d) and after (e, f) corrosion. In c) and e), the enlarged areas shown in d) and f) are indicated by dashed squares; moreover, the original boundaries of the wire are marked by the dashed lines in e).

Comparison of XP spectra of the neat $[C_6C_1Im][Br_2I]$, $[C_6C_1Im]I$, and $[C_6C_1Im]Br$ ILs

The spectra of the trihalide ionic liquid and the neat bromide and iodide ILs are presented in Figure S2; binding energy values of all signals are referenced to the position of the hexyl group signal C_{alkyl} at 284.8 eV.

Most obviously, the I and Br signals from the $[Br_2I]^-$ polyhalide anions are found at considerably higher binding energies relative to the ones from single halides ILs: the trihalide I $3d_{5/2}$ peak is shifted by +2.4 eV and the Br $3d_{5/2}$ peak by +1.1 eV to higher binding energy, compared to $[C_6C_1Im]I$ and $[C_6C_1Im]Br$, respectively, fully in line with the results from Men *et al*.[1] These upwards shifts reflect the spreading of the negative charge across the three centres in the trihalide anion, in contrast to its localisation at the I^- and Br^- anion in $[C_6C_1Im]I$ and $[C_6C_1Im]Br$, respectively.

In addition, the slight upward shifts (about +0.2 eV) of signals originating from the cationic imidazolium ring (C 1s: C_{hetero} and N 1s: N_{cation}) when paired with $[Br_2I]^-$ compared to the single halides reveal the relatively low basicity of the trihalide anion.[1]

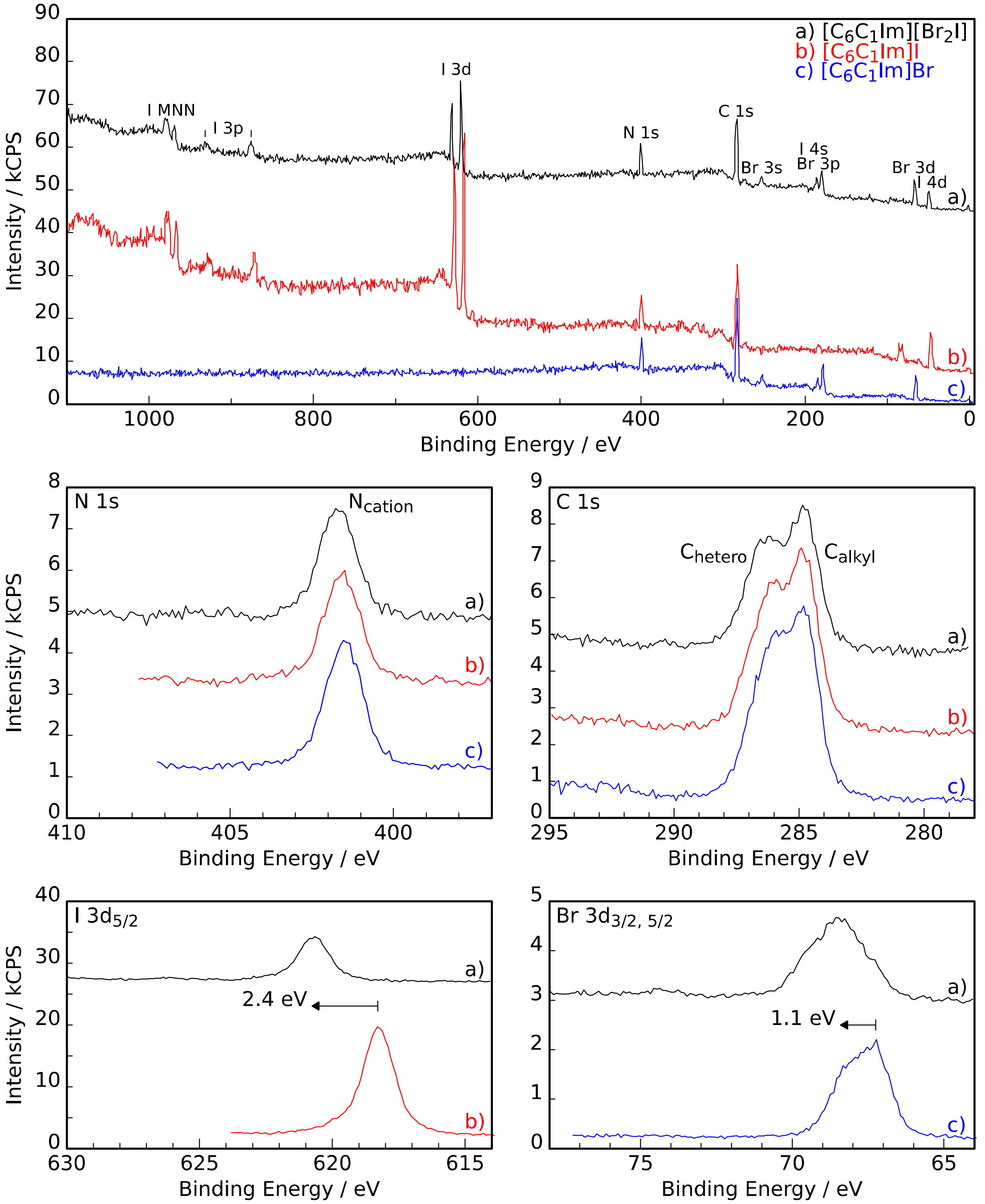

Figure S2: XP survey (top) and detailed spectra of the neat $[C_6C_1Im][Br_2I]$ (black, a), $[C_6C_1Im]I$ (red, b) and $[C_6C_1Im]Br$ (blue, c) for direct comparison. All spectra are referenced to aliphatic carbon C_{alkyl} at 284.8 eV.

Vapour phase mass spectroscopy

To test for volatile components during metal corrosion under vacuum conditions, gas phase spectra of $[C_6C_1Im][Br_2I]$ in contact with copper and with molybdenum were recorded in a separate small system for mass spectrometry (Mass Spectrometry Chamber, MSC, base pressure: 5×10^{-8} mbar). Copper and molybdenum were chosen as the extreme cases of most reactive vs. least reactive metals with $[C_6C_1Im][Br_2I]$, respectively. The MSC is a small UHV chamber equipped with a copper sample head that can be directly cooled and heated, and a quadrupole mass spectrometer (QMS, Hiden HAL 511 3F, 2–550 amu, for details see Deyko *et al.*[2]).

A small drop of $[C_6C_1Im][Br_2I]$ was spread onto a copper foil covering about 10 x 10 mm^2. Upon contact with the copper, the $[C_6C_1Im][Br_2I]$ film turned black within one minute. The sample was quickly put into the vented MSC and pumping was started. In contrast to conventional non-trihalide ILs, the decrease in pressure was observed to be much slower (typically after 30mins a pressure of 10^{-5} mbar is easily obtained with this setup). After 2.5h from the start of pump down, the chamber eventually reached a pressure below 10^{-5} mbar that allowed us to the start the QMS in a very insensitive detection mode – that is, a low acceleration voltage of the secondary electron multiplier detector – for safety reasons (note that signal-to-noise ratio is thus very low for small signals), and gas phase mass spectra were taken (example shown by red spectrum b in Figure S3). Visual inspection 24 hours later at a chamber pressure of 10^{-7} mbar revealed that the colour of the IL film on the copper plate had considerably lightened compared to the black colour at the beginning. A mass spectrum after 24 hours degassing (using high QMS-sensitivity with much better signal-to-noise ratio) is also shown in Figure S3 (blue spectrum a) along with a mass spectrum of a $[C_6C_1Im][Br_2I]$ film sample spread on a molybdenum foil (black spectrum c in Figure S3, after about 2h pump down; high QMS-sensitivity). Note, the individual spectra regions are multiplied with very different scaling factors for direct comparison on a linear scale (see Figure S3). During pumpdown (red spectrum b) the sample off-gassed a significant amount of iodine, which can be seen as I^+ and I_2^+ mass signals peaks. The I^+ peak is significantly more prominent that the I_2^+, as expected for gas phase I_2 due to cracking inside the ionisation source of the QMS.[3] The intensity of these I_2-related signals decreased by about two orders of magnitude after 24h pumping. In addition to the I_2^+ and I^+ signals, we

also detected HI^+, which is attributed to reaction with residual H_2 and H_2O in the gas background of our MSC-System in the QMS ionisation source: similar HI^+ signals along with I_2^+ and I^+ were also detected when inserting small amounts of I_2 vapour via a gas valve into the MSC chamber.

Notably, no significant amount of gas phase Br_2 could be detected during this experiment, neither in the Br_2^+ region (which is the most prominent signal for gas phase Br_2 [4]) nor in the Br^+ region, see Figure S3. The same holds true for IBr^+, which was not detected either (spectral region not shown).

As a second experiment, a small drop of $[C_6C_1Im][Br_2I]$ was spread onto a clean molybdenum foil, which was loaded into the MSC and pumped down. Since the pump down time in this case was similar to non-trihalide ILs, the QMS could be started after about two hours in the high-sensitivity mode. The mass spectra collected at room temperature (black spectrum c in Figure S3) and at 40 °C elevated spectra (not shown) did not exhibit significant I_2-related signals in the gas phase; the small I^+/HI^+ peak is assigned to a background contamination from the previous experiments, because it is visible even without a sample loaded.

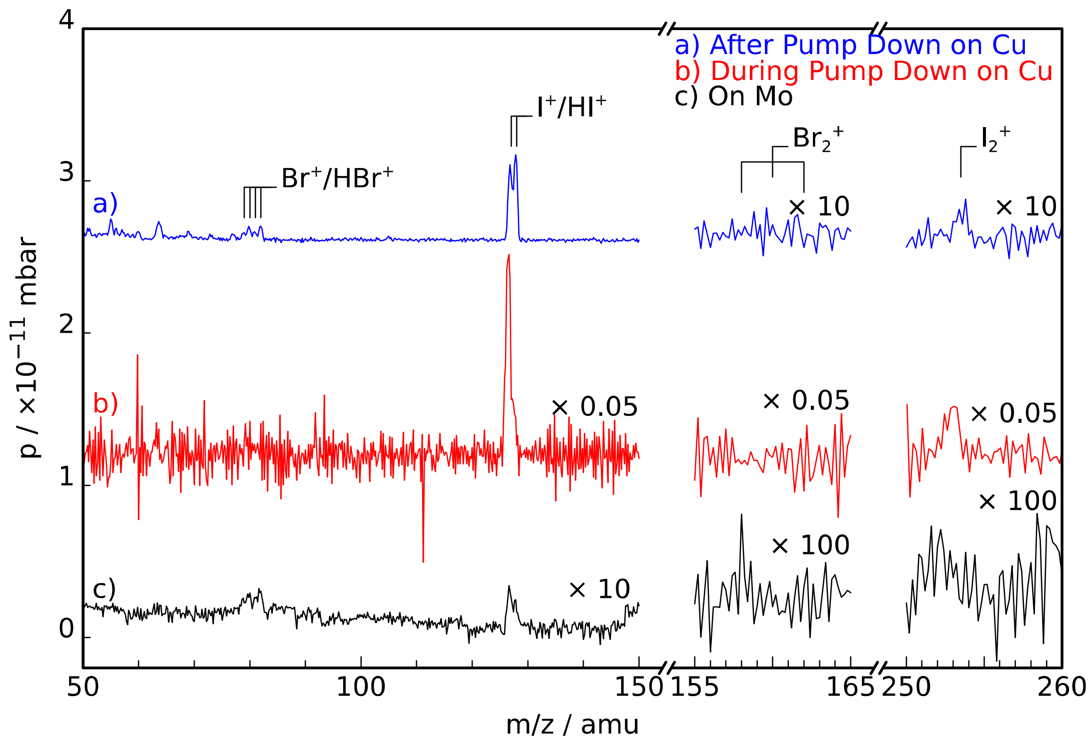

Figure S3: Gas phase QMS analysis for $[C_6C_1Im][Br_2I]$ film samples spread on pre-cleaned polycrystalline copper and molybdenum foils: blue spectrum (a) after 24 h pump down on Cu, red spectrum (b) after only 2.5 h pump down on Cu, and black spectrum (c) after 2 h

pump down time on Mo. Note that individual regions are re-scaled and off-set for sake of clarity. Further details are given in the text above.

Corrosion of gold by $[C_4C_1C_1Im][Br_2I]$, dry $[C_6C_1Im][Br_2I]$ and wet $[C_6C1Im][Br_2I]$

Table S1: Change in the mass of gold foils after corrosion by dry $[C_6C_1Im][Br_2I]$, by water saturated (wet) $[C_6C_1Im][Br_2I]$ and by $[C_6C_1Im][Br_2I]$ under standard ambient conditions for 6h at 40°C, along with the data after corroding gold by the trihalide IL $[C_4C_1C_1Im][Br_2I]$ methylated in C2-position (last column). Corresponding Au 4f spectra of the solutions are shown in Figure S4.

	Dry	Wet	Ambient	Methylated
Initial / mg	609	526	480	620
Final / mg	361	287	245	445
Δ / mg	248	238	235	175
Δ / mmol	1.26	1.21	1.19	0.887
dissolution rate / $g{\cdot}dm^{-2}{\cdot}d^{-1}$	15.7	17.5	18.9	10.9
etch rate / $\mu m{\cdot}min^{-1}$	0.057	0.063	0.068	0.039
IL / mg	2072	1933	1783	1422
IL / mmol	4.56	4.26	3.93	3.23
Metal:Im	0.28:1	0.28:1	0.30:1	0.27:1

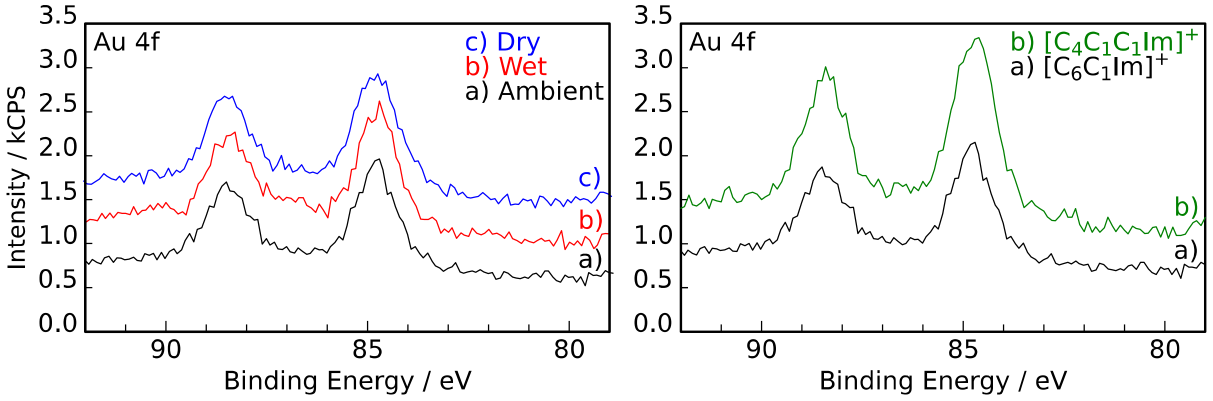

Figure S4: Left panel shows the Au 4f region of IL solutions after exposing a gold foil for 6h at 40°C under standard ambient (black, a), water-saturated wet (red, b), and dry (blue, c) conditions to $[C_6C_1Im][Br_2I]$ (for details, see text). Right panel: Au 4f region after exposing a gold foil for 6h at 50°C to the C2-methylated IL $[C_4C_1C_1Im][Br_2I]$ (green, b) in comparison to the solution after corroding gold for 6h at 40°C to $[C_6C_1Im][Br_2I]$ (black, a), both under ambient conditions.

^{1}H-NMR and ^{13}C-NMR of $[C_6C_1Im][Br_2I]$

The sample denoted as Au-21h in Table 1 (extended gold corrosion experiment) and a sample of the same batch of the neat $[C_6C_1Im][Br_2I]$ IL was subjected to ^{1}H-NMR (Figure S5) and ^{13}C-NMR (Figure S6). Both, ^{1}H and ^{13}C spectra of the $[C_6C_1Im]^+$ cation (proton and carbon assignments of signals shown in insets of Figure S5 and S6) before and after corrosion do not exhibit significant changes. In particular, the signals of the most acidic proton and the carbon in 2-position at around 7.8 ppm and 137 ppm, respectively, do not show any decrease in intensity after corrosion as it would be expected if proton abstraction forming an imidazole-carbene species was occurring during the corrosion reaction.

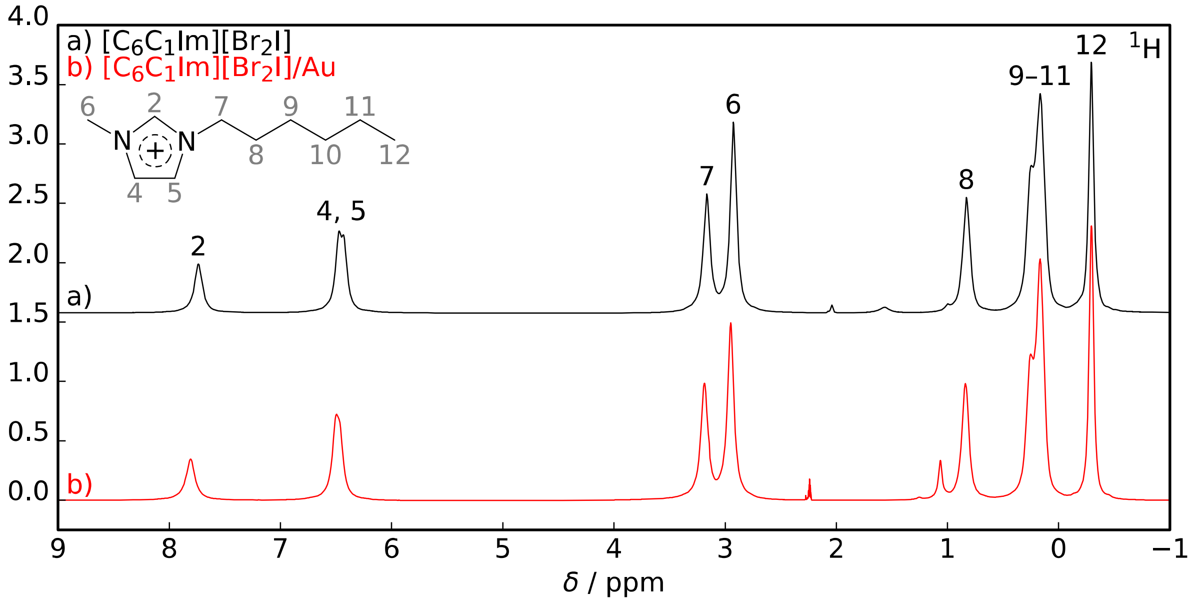

Figure S5: ^{1}H-NMR spectra of neat $[C_6C_1Im][Br_2I]$ (black spectrum a) and of the solution after having reacted $[C_6C_1Im][Br_2I]$ with gold over 21 hours (red spectrum b, for details, see main text). Acquired spectra were recorded without co-solvent with an acetone standard as a sealed insert seen at around 2ppm. Note that the red spectrum is shifted by +0.20 ppm to align the signal 12 from the hexyl chain's terminal methyl group, and is vertically off-set for direct comparison.

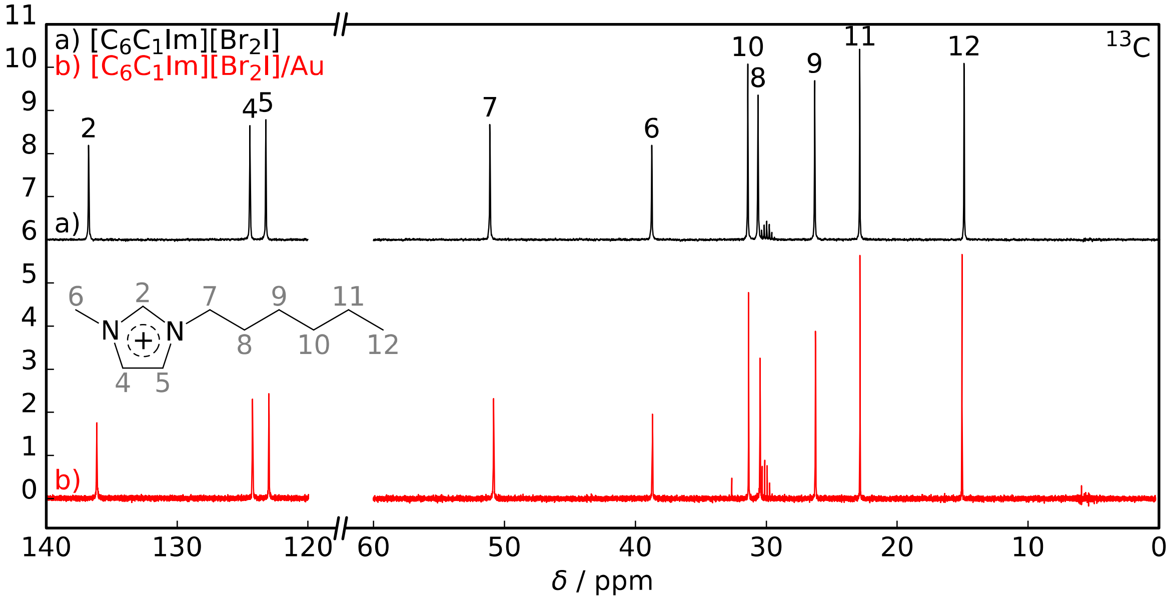

<u>Figure S6:</u> ^{13}C-NMR spectra of neat [C_6C_1Im][Br_2I] (black spectrum a) and of the solution after having reacted [C_6C_1Im][Br_2I] with gold over 21 hours (red spectrum b, for details, see main text).

References

[1] S. A. Men, K. R. J. Lovelock, P. Licence, *Chem. Phys. Lett.* **2017**, *679*, 207-211.

[2] A. Deyko, S. Bajus, F. Rietzler, A. Bösmann, P. Wasserscheid, H. P. Steinrück, F. Maier, *J. Phys. Chem. C* **2013**, *117*, 22939-22946.

[3] M. Kundel, R.-J. Huang, U. R. Thorenz, J. Bosle, M. J. D. Mann, M. Ries, T. Hoffmann, *Anal. Chem.* **2012**, *84*, 1439-1445.

[4] S. E. Stein (director), "Mass Spectra" in *NIST Chemistry WebBook, NIST Standard Reference Database Number 69* (Eds.: P. J. Linstrom, W. G. Mallard), NIST Mass Spec Data Center, Gaithersburg MD, 20899, doi: 10.18434/T4D303, retrieved February 12, 2018.
